# Drug therapy problems and contributing factors in the management of heart failure patients in Jimma University Specialized Hospital, Southwest Ethiopia

**DOI:** 10.1371/journal.pone.0206120

**Published:** 2018-10-23

**Authors:** Yirga Legesse Niriayo, Kabaye Kumela, Tesfaye Dessale Kassa, Mulugeta Tarekegn Angamo

**Affiliations:** 1 Department of Clinical Pharmacy, School of Pharmacy,College of Health Sciences, Mekelle University, Mekelle, Tigray, Ethiopia; 2 Department of Clinical Pharmacy, School of Pharmacy, Faculty of Health Sciences, Jimma University, Jimma, Oromyia, Ethiopia; 3 Division of Pharmacy, School of Medicine, University of Tasmania, Hobar, Australia; University of Auckland, NEW ZEALAND

## Abstract

**Background:**

Drug therapy problem (DTP) is any unwanted incident related to medication therapy that actually or potentially affects the desired goals of treatment. Heart failure (HF) patients are more likely to experience DTP owing to multiple prescriptions and comorbidities. Despite the serious negative impact of DTP on treatment outcomes, there is a dearth of study on DTP among HF patients in Ethiopia.

**Objective:**

The main aim of this study was to assess the prevalence and contributing factors of DTP among ambulatory HF patients in Jimma University Specialized Hospital, Ethiopia.

**Methods:**

A hospital based prospective observational study was conducted. Written informed consent was obtained from each patient after full explanation of the study. Data were collected through patient interview and expert review of medical, medication and laboratory records of one-year follow-up from May 2015 to April 2016. DTPs were identified using Cipolle’s method followed by consensus review with experts. Binary logistic regression was performed to identify factors contributing to DTP. A p<0.05 was considered statistically significant in all analyses.

**Result:**

Of 340 study participants; male to female ratio was equivalent, the mean (± SD = standard deviation) age was 50.5±15.6 years. Eight hundred eighty DTPs were identified equating 2.6 ±1.8 DTPs per patient. The frequently identified DTPs were dosage too low (27.8%), ineffective drug therapy (27.6%) and need additional drug therapy (27.4%). Most commonly implicated drugs were beta-blockers (34.4%), angiotensin converting enzyme inhibitors (24.8%), statins (16.5%) and antithrombotics (13.1%). Factors contributing to DTP were age >50 years (AOR [adjusted odd ratio] = 5.43, 95%CI [95% confidence interval] = 2.03–14.50); negative medication belief (AOR = 3.50, 95%CI = 1.22–10.05); poor involvement of patients in the therapeutic decision makings (AOR = 4.11, 95%CI = 1.91–8.88); number of co-morbidity≥2(AOR = 5.26, 95%CI = 2.38–11.65) and number of medications ≥5 (AOR = 3.68, 95%CI = 1.28–10.51).

**Conclusion:**

DTPs are common among ambulatory care HF patients. Patients with older age, negative medication belief, polypharmacy, co-morbidities and those who were poorly involved in the therapeutic decision were more likely to experience DTP. Despite traditional prescription refilling, an integrated multidisciplinary approach involving patients and clinically trained pharmacists should be implemented in the patient care process at ambulatory care clinics in order to improve overall outcomes and reduce DTPs and associated burdens in HF patients.

## Background

Heart failure (HF) is a progressive and complex clinical syndrome that can occur due to impairment of cardiac structure or function that leads to the inability of the heart to fill with or pump sufficient amount of blood to meet the metabolic need of the body [1–3]. HF is a major public health problem that affects about 26 million peoples worldwide [4,5]. In spite of effective pharmacotherapy, HF remains the leading cause of morbidity, mortality and economic burden for health care budgets [6, 7].

Heart failure management often involves lifestyle modification and lifelong therapy with multiple medications, and the list of medications has expanded considerably since the last three decades [8]. HF treatment has been largely an add-on phenomenon; thus, optimal HF therapy has become increasingly complex [8, 9]. According to evidence-based guidelines, angiotensin-converting enzyme inhibitors (ACEIs), beta-blockers (BBs), angiotensin receptor blockers (ARBs), hydralazine with long acting nitrates, and spironolactone have all been shown to decrease morbidity and mortality in HF [1,3,10]. Despite this, medications for HF are often underutilized [10–12].

Drug therapy problem (DTP) refers to any unwanted incident related to medication therapy that actually or potentially affects the desired goals of treatment [13,14]. DTP can occur at all steps of the treatment process, mainly during prescribing, transcribing, dispensing and patient use of medication therapy [15]. DTPs are categorized into seven major classes. These include; unnecessary drug therapy, need additional drug therapy, ineffective drug therapy, dosage too low, dosage too high, adverse drug reaction (ADR) and non-compliance [14].

Drug therapy problem is one of the public health problems worldwide and has been significantly increased over the past few decades [16]. It was estimated that around 5–10% of hospital admissions were due to DTPs, in which more than half of them are avoidable [16]. Worldwide, more than half of all medicines are prescribed and dispensed inappropriately and half of the patients fail to take them correctly [17,18]. Although most medications are beneficial with excellent safety profile, DTPs may compromise the quality of life, increase hospitalization, increase overall health care cost and even leads to death if not detected and resolved early [19].

Drug therapy plays a crucial role in the treatment and improvement of the well-being of HF patients. However, the benefits of drugs brought to patients may be compromised due to the occurrence of DTPs. DTPs are frequent in HF patients mainly owing to multiple prescriptions, multi-comorbidities, and advanced age [8,20,21]. In patients with HF and other cardiovascular diseases, the frequency of DTPs has been reported to be as high as 78% and 69%, respectively [22, 23]. The majority of preventable HF hospitalizations, deaths and increased health care cost are due to poor adherence to the use of approved evidence-based medications [24]. It has been reported that about 17% of HF patients may experience adverse effects of their medication and ADR-related hospitalizations account for 6.7% of admissions of HF patients to cardiac units [25].

There are limited studies published on the type and extent of DTPs and their contributing factors in developing countries [26]. To our knowledge, there is no study done on DTP among HF patients at ambulatory care setting in Ethiopia. Hence, it is very crucial to conduct studies on DTPs in ambulatory care HF patients in countries like Ethiopia, where there are limited healthcare resources, overburdened health care system and inadequate trained health care professionals, in order to develop preventive strategies. Therefore, this study was aimed to assess the magnitude of DTPs and their contributing factors in the management of patients with HF in ambulatory care clinic of Jimma University Specialized Hospital (JUSH), Southwest Ethiopia.

## Methods and materials

### Study design and study setting

A hospital based prospective observational study was conducted at the ambulatory care clinic in JUSH, Oromia, Ethiopia. JUSH is a teaching and referral hospital. It is the largest public hospital in southwest Ethiopia which is estimated to have about 15 million catchment population.

### Study population and data collection procedure

Consented HF patients aged ≥ 18 years who had complete medical records and regular follow-up between May 2015 and April 2016 were included in the study. Patients were recruited into the study during their appointment for medication refilling. Patients were excluded if they were newly diagnosed with HF (<6 months), seriously ill to complete the interview, unwilling to give consent, and their medical record not complete or available for further review.

A sample of 355 was calculated using a single population proportion formula assuming 50% proportion of DTPs, 95% confidence level, 5% margin of error and 10% of contingency for non-response rate. We approached a total of 355 participants; of whom, 15 patients were excluded from the study due to the duration of HF<6 month (3), unwilling to give consent (4), and incomplete medical record (7) and serious illness to complete the interview (1). Patients were interviewed consecutively according to their appointment schedule using the interview questionnaire and their respective medical chart was retrieved using the retrieval checklist. Interview questions included socio-demographics, medication adherence, medication experience and previous medication history assessment. Respective patient’s medical, medication and laboratory records were reviewed. Three clinical pharmacists, two nurses and one physician were involved in data collection. Training and orientation were given to professionals involved in data collection.

### Drug therapy problems identification and assessment

Drug therapy problems were identified and classified using the Cipolle’s method [14] followed by a consensus meeting with a panel of experts including medical internists and clinical pharmacists. The experts further refined DTP identification and classification method to the study setting based on treatment guidelines and literature reviews [1,3]. The identification of DTP was based on a review of patients’ medical and medication records, assessment of laboratory investigations, patients interview about medication experience, and physical observation. Specific information about medication therapies, such as the recommended drug of choice, recommended dosages, frequency of administration, duration of therapy, drug-interactions and adverse drug events were compared based on details from the standard pharmacotherapy textbooks and HF treatment guidelines [1–3].

### Definition of terms and variables

Participants who were able to read and write, and had attended at least to an elementary school level were said to be educated, otherwise, they were considered as uneducated. Poly-pharmacy refers to the concurrent use of greater than or equal to five drugs. Patient belief on medication refers to the patient's attitude toward taking medication and description of wants, concerns, understanding, beliefs, and behaviours which was measured by belief about medication questioner (BMQ), in which the patient’s belief was considered as positive when the average sum of 5-item patient’s medication necessity scale score exceeded the average 5-item medication concerns scale otherwise it was considered as negative. Patient involvement in drug therapy decision means the degree of involvement of patients in the decision of their drug therapy, which was measured using 2-item 5-point Likert scale using self-reported questionnaire about patient involvement in therapeutic decision, where respondents indicated their degree of involvement ranging from 1 = never to 5 = all times. Accordingly, patients were said to be actively involved if the average score was above the mid-point (>5) otherwise poorly involved. Medication availability means the pattern of availability of medicines in the healthcare institution, where the participants get services, which was measured using single item self-reported questionnaire that contains a 5-point Likert scale question where respondents indicated their degree of agreement about the availability of their medicines ranging from 1 = never to 5 = all times. Accordingly, patients were said to have no availability problem if the patient responded their medicines were available most of the time /all times but if they responded their medicines were available sometimes/rarely/never, then it was considered that they had availability problem. DTP refers to any unwanted incident related to medication therapy that actually or potentially affects the desired goals of treatment, which was identified using Cipolle’s method [14] followed by a consensus meeting with experts. DTPs were categorized in to seven major groups base on Cipolle’s method. These include: including unnecessary drug therapy, needs additional drug therapy, ineffective drug therapy, dosage too low, adverse drug reaction, dosage too high and noncompliance.

Category of DTPs were defined and identified based on Cipolle’s method [14] as follows:

Unnecessary drug therapy is considered when:

- there is no valid medical indication for the drug therapy at this time,
- multiple drug products are being used for a condition that requires single drug therapy,
- the medical condition is more appropriately treated with nondrug therapy
- the drug therapy is used to treat an avoidable adverse reaction associated with another medication.

Need for additional drug therapy was considered when:

- the medical condition requires the initiation of drug therapy
- Preventive drug therapy is required to reduce the risk of developing a new condition.
- the medical condition requires additional drug therapy to achieve synergistic or additive effects.

Ineffective drug therapy was considered when:

- the least effective drug is used while the most effective drug is available
- the drug is used for medical condition which is refractory to the drug product.
- the drug product used is not an effective product for the medical condition being treated

Dosage too high was considered when:

- the dose is too high
- dosing frequency is too short
- the duration of drug therapy is too long
- the drug interaction occurs that could result in a toxic reaction to the drug product
- the dose of the drug was administered too rapidly

Dosage too low was considered when:

- the dose of the drug is too low to produce the desired response
- the drug interaction occurs that could reduce the amount of active drug available
- the duration of drug therapy is too short to produce the desired response
- the dosage interval is too infrequent to produce the desired response

Adverse drug reaction was considered when:

- the drug causes an undesirable reaction that was not dose-related
- the drug interaction causes an undesirable reaction that is not dose-related
- the drug causes an allergic reaction
- the drug is contraindicated due to risk factors
- the dosage regimen administered or changed too rapidly

Noncompliance is considered if a patient fails to take medications appropriately due to one of the following reasons:

- lack of understanding the instructions
- preference not to take the medication
- forgetfulness
- inability to swallow or self-administer the drug product appropriately
- affordability and availability problem

### Data analysis

Data were entered into an EPI data management and analyzed using the IBM Statistical Package for the Social Science (IBM SPSS version 20.0 Inc., Chicago, Illinois). Descriptive analysis was computed as frequency, mean and standard deviation (SD). Multicollinearity was checked to test correlation among predictor variables using variance inflation factor and none was collinear. Univariable logistic regression analysis was performed to determine the association of each independent variable with DTP, and then independent variables with p value <0.25 in univariable analysis were included into a multivariable binary logistic regression model to identify predictors of DTP. A p value of <0.05 was considered statistically significant in all analyses.

### Ethics

This study was approved by the institutional review board (IRB) of Jimma University, College of Health Sciences. The purpose and protocol of this study was fully explained to all study participants. Written informed consent was obtained from all participants included in this study. The privacy of personal information was entirely confidential and protected. All the methods were performed in accordance with approved institutional guidelines.

## Results

### Socio-demographic characteristics

A total of 355 patients were approached; of whom, 340 patients were included in the study while 15 patients were excluded from the study due to duration of HF<6 months (3), unwilling to give consent (4), and incomplete medical record (7) and serious illness to complete the interview (1). Out of the total, 171 (50.3%) were females and the mean (± SD) age was 50.5±15.6 years. Majorities, 214 (62.9%) were from rural and more than half of them, 194 (57.1%) were not educated. With regard to social drug use, 7.9% and 10.3% of the participants were alcohol consumers and khat chewers, respectively. All of the participants were non-smokers. Two-hundred nineteen (64.4%) of the participants had a positive belief about their medication (Table 1).

**Table 1:** Sociodemographic characteristics of HF patients in JUSH, 2016 (n = 340).

Characteristics	n (%)
Gender	
Female	171 (50.3)
Male	169 (49.7)
Age in years,	
< = 50	178 (52.4)
>50	162 (47.6)
Mean ± SD	49.8 ± 16.2
Median (IQR)	50 (38–61)
Residence,	
Rural	214 (62.9)
Urban	126 (37.1)
Educational level	
Uneducated	194 (57.1)
Educated	146 (42.9)
Social drug users,	
Alcohol consumers	27 (7.9)
Khat chewers	35 (10.3)
Traditional medicine users	55 (16.2)
Living status	
Living with family/friends	315 (92.7)
Living alone	25 (7.4)
Medication belief	
Positive belief	219 (64.4)
Negative belief	121 (35.6)

### Clinical, medication and health care related characteristics

The mean (±SD) number of comorbidities per patient was 1.9±0.9. The commonly identified comorbidities in HF patients were ischemic heart diseases (43.5%), hypertension (25%), valvular heart diseases (21.2%) and atrial fibrillation (20.9%). The mean(±SD) duration of HF follow-up period was 2.6±2.1years. The majority of patients were in New York Heart Association (NYHA) classes III (48.5%) followed by class II (42.7%). More than half of the patients had been hospitalized one or more times within one-year data retrieval period. A total of 1,757 medication orders containing 1,389 medications were reviewed. The average number of medications per patient was 4.1±1.3. The most commonly prescribed drugs were diuretics (73.2%) followed by ACEIs (71.2%) and BBs (59.1%. Only 92 (27.1%) patients were actively involved in their drug therapy decision, and 112 (32.9%) patients had availability problems of their medicines in the health institution where they had a follow-up for treatment (Table 2).

**Table 2:** Clinical, medication and healthcare related characteristics associated with ambulatory care HF patients in JUSH, 2016 (n = 340).

Characteristics	n (%)
Mean (±SD) number of comorbidities	1.9 ± 0.9
Major comorbidities	
Ischemic heart diseases	148 (43.5)
Hypertension	85 (25)
Valvular heart diseases	72 (21.2)
Atrial fibrillation	71 (20.9)
Commonly used medications	
Loop diuretics	249 (73.2)
ACEIs	242 (71.2)
BBs: Proven BB*	55 (16.2)
Atenolol**	146 (42.9)
Antiplatlets	198 (58.2)
Statins	142 (41.8)
Proton pump inhibitors	80 (23.5%)
Potassium sparing diuretics	76 (22.4)
Cardiac glycosides	63 (18.5)
Patient involvement in decision of drug therapy	
Actively involved	92 (27.1)
Poorly involved	14 8 (72.9)
NYHA class	
Class I	18 (5.3)
Class II	145 (42.7)
Class III	165 (48.5)
Class IV	12 (3.5)
Heart failure based on etiologic origin	
Ischemic etiology	138 (40.6)
Non-ischemic etiology	202 (59.4)
Number of hospitalizations since the last year	
Never	138 (40.6)
One or more time	202 (59.4)
Number of medicines per patient	
<5 medication	211 (62.1)
≥5 medication	129 (37.9)
Medication availability problem	
No	228 (67.1)
Yes	112 (32.9)

*Beta-blockers whose use in HF is proven in randomized controlled clinical trials (metoprolol, carvedilol)

** the use of atenolol in HF is not proven in randomized clinical trial

### Prevalence of drug therapy problems

Eight-hundred eighty DTPs were identified with a mean (± SD) DTP per patient of 2.6±1.8. One or more DTPs have been identified in 83.5% of the patients. The frequently identified DTPs were dosage too low (27.8%), ineffective drug therapy (27.6%), and need additional drug therapy (27.4%) (Table 3).

**Table 3:** Types and causes of DTPs among ambulatory care HF patients in JUSH, 2016.

Types and causes of DTP	Proportions with regard to patients (n = 340), n (%)	Proportions with regard to DTPs (n = 880), n (%)
**Unnecessary drug therapy**		
No medical condition	24 (7.1)	24 (2.7)
Duplicate therapy	6 (1.8)	7 (0.8)
**Need additional drug therapy**		
Untreated indication/condition	112 (32.9)	163 (18.5)
Preventive or prophylactic	64 (18.8)	78(8.9)
**In-effective drug therapy**		
More effective drug available	175 (51.5)	239 (27.2)
Not effective for the condition	4 (1.2)	4 (0.4)
Dosage too low	198 (57.7)	245 (27.8)
**Unsafe drug therapy**		
Adverse drug reaction	26 (7.6)	26 (3)
Dosage too high	14 (4.1)	14 (1.6)
**Non-compliance**		
Not remember to take the medication	50 (14.7)	50 (5.3)
Not take the medication during recovery	18 (5.3)	18 (2.3)
Not take the medication during exacerbation	12 (3.5)	12 (1.4)

### Drugs commonly implicated in DTPs

**Fig 1:**
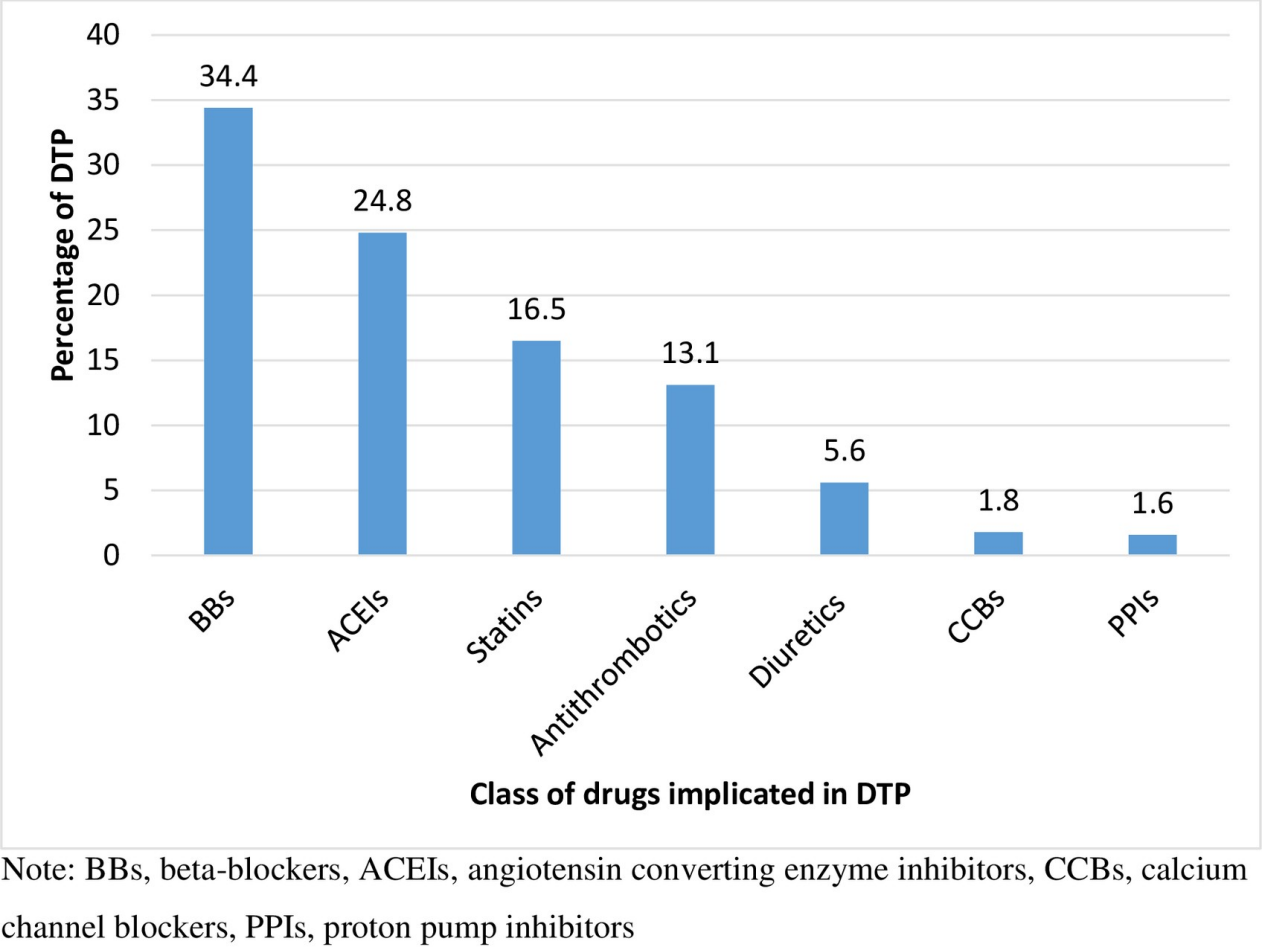
Commonly implicated class of drugs in DTPs in ambulatory HF patients in JUSH,2016.

The commonly implicated classes of drugs in DTPs were BBs (34.4%) followed by ACEIs (24.8%), statins (16.5%) and antithrombotic (13.1%) (Fig 1). Overall, 61% of DTPs were due to HF medications. BBs were frequently involved in ineffective drug therapy, dosage too low and need additional drug therapy while ACEIs were commonly associated with dosage too low. Although atenolol is not evidence-based BB for treating HF, it was prescribed for 146 (42.9%) patients. Only 55 (16.2%) of the patients were on evidence-based BBs. On the other hand, 84 (24.7%) of the patients were in need of BBs. Statins and antithrombotic were commonly associated with need additional drug therapy and ineffective drug therapy.

### Factors contributing to DTPs

Univariable logistic regression analysis was performed to compare HF patients with DTP and without DTP using the socio-demographic, disease and medication related characteristics. Accordingly, more proportion of male patients than females had DTP (52.1% vs. 47.9%, p = 0.048). More proportion of older (age > 50 years) patients than younger patients developed DTPs (54.2% vs. 45.8%, p<0.001). More proportion of patients with a negative belief about their medication than those with a positive belief experienced DTP (59.2% vs.40.8%, p = 0.037). Patients actively involved in medication decision-making developed less DTP than patients poorly involved (21.5% vs. 78.5%, p<0.001). Patients with higher number of comorbidity and medication had more DTPs than patients with less co-morbidity and medication (Table 4).

**Table 4:** Factors contributing to DTPs among ambulatory HF patients in JUSH, 2016 (n = 340).

	DTP		Bivariable analysis			Multivariable analysis		
	No, n (%)	Yes, n (%)	B	COR (95% CI)	P-value	B	AOR (95%CI)	P-value
Gender, male	21 (37.5)	148 (52.1)	0.59	1.81(1.01–3.26)	0.048	0.03	1.03 (0.47–2.27)	0.937
Age category								
< = 50	48 (85.7)	130 (45.8)	1	1	1	1	1	1
>50	8 (14.3)	154 (54.2)	1.96	7.11(3.25–15.57)	<0.001	1.69	5.43 (2.03–14.50)	0.001
Residence, rural	30 (53.6)	184 (64.8)	0.47	1.59 (0.89–2.85)	0.114	0.17	1.18 (0.56–2.51)	0.663
Traditional medicine users	6 (10.7)	49 (17.3)	0.55	1.74 (0.71–4.28)	0.229	-0.09	0.91 (0.28–3.02)	0.881
Belief about medication								
Positive belief	5 (8.9)	116 (40.8)	1	1	1		1	1
Negative belief	51(91.1)	168 (59.2)	1.95	7.04 (2.73–18.18)	<0.001	1.25	3.50 (1.22–10.05)	0.020
Involvement in therapeutic decision								
Actively involved	31 (55.4)	61(21.5)	1	1	1	1	1	1
Poorly involved	25 (44.6)	223 (78.5)	1.51	4.53 (2.49–8.25)	<0.001	1.41	4.11 (1.91–8.88)	<0.001
Medication availability problem								
No	44 (78.6)	184 (64.8)	1	1	1	1	1	1
Yes	12 (21.4)	100 (35.2)	0.69	1.99 (1.01–3.95)	0.048	0.20	1.22 (0.50–2.99)	0.667
Hospitalization in the preceding one year								
Never	30 (53.6)	118 (41.5)	1	1	1	1	1	1
≥ one times	26 (46.4)	166 (58.5)	0.48	1.62 (0.91–2.89)	0.099	0.07	1.07 (0.48–2.40)	0.872
Etiology of HF								
Ischemic origin	7 (12.5)	131 (46.1)	1.79	5.99 (2.63–13.69	<0.001	0.84	2.31 (0.87–6.14)	0.093
Non-ischemic origin	49 (87.5)	153 (53.9)	1	1	1	1	1	
Duration with HF (year)	2.65±2.05		0.21	1.24 (1.04–1.47)	0.016	0.15	1.17 (0.94–1.45)	0.157
Number of comorbidities ≥2	14 (25.0)	195 (68.7)	1.88	6.57 (3.41–12.65)	<0.001	1.66	5.26 (2.38–11.65)	<0.001
Number of medications ≥5	5 (8.9)	124 (43.7)	2.06	7.91(3.08–20.40)	<0.001	1.30	3.68 (1.28–10.51)	0.015

DTP-Drug therapy problem, CI-confidence interval, COR-Crude odds ratio, AOR-adjusted odds ratio, HF-heart failure

Variables with P<0.25 in the univariable analyses were included in the multivariable logistic regression model. The whole model containing all predictors was statistically significant (Chi-square = 113.94, df = 12, P<0.001). According to multivariable analysis; age >50 years (AOR = 5.43, 95%CI = 2.03–14.50), negative belief on medication (AOR = 3.50, 95%CI = 1.22–10.05), poor involvement of patients in therapeutic decision making (AOR = 4.11, 95%CI = 1.91–8.88), number of co-morbidities ≥2 (AOR = 5.26, 95%CI = 2.38–11.65) and number of medication ≥5 (AOR = 3.68, 95%CI = 1.28–10.51) were found to be contributing factors to DTPs (Table 4).

## Discussion

Clinicians may not be aware of the actual burden of DTP and its contributing factors. Hence, identification and documentation of DTP and its predictors will help them to design preventive strategies of DTP and provide responsible care of patients [14]. Therefore, our study determined the prevalence of DTPs and identified contributing factors and drugs implicated in DTP at ambulatory care of JUSH, Southwest Ethiopia. We have identified at least one DTP in 83.5% of study participants, which was in line to other studies [21, 23, 27–29]. The mean number of DTPs per patient was 2.60, which was lower than the mean reported in Taiwan, 5.90 DTPs per patient [30], but higher than that of Spanish study, 1.5 DTP/patient [21]. This might variation might be due to the differences in a study design, clinical characteristics, population demographics, medication therapy used and methods of DTP identification and classification.

In agreement to the study conducted in Spain [21], dosage too low, ineffective drug therapy and need additional drug therapy were frequently identified DTPs. In contrary to the study from Thailand [28] and review of observational studies [31], where the rate of adherence ranged from 40–60%, noncompliance and unnecessary drug therapy were reported to be low. The possible reason for this variation could also be due to the difference in methods of adherence measurement, medical practitioner’s expertise and experiences, disease distribution and population demographics.

In contrary to Taiwan [30] study, BBs and statins were frequently implicated drug classes in DTPs. This might be attributed to lack of awareness of medical practitioners in our settings with regard to superior role of evidence-based BBs and high-intensity statins over atenolol and low intensity statins, respectively, which were the major contributors of ineffective drug therapy in the current study.

Evidence-based guidelines [1, 3] recommend the use of only 3 BBs (metoprolol, carvedilol, and bisoprolol) that have proven survival benefit in HF patients. However, atenolol was prescribed in 42.9% of patients in this study. In addition, ACEIs and BBs are recommended for all patients with systolic HF unless contraindicated [1, 3]. In contrast, 24.7% and 5.3% of the patients were not taking BBs and ACEIs, respectively, while they were eligible for therapy. These all could be attributed to the absence of HF management guideline in our settings.

In the current study, about 61.0% of DTPs were due to HF medications. This is inconsistent with a study in Spain [21], in which majority of DTPs (78%) were due to non-HF medications like anti-diabetics and allopurinol. This might be due to variation in diseases distribution and drug therapy used. Similar to previous studies [21, 23, 32–35], patients with polypharmacy (≥5 drugs) were approximately three-times more likely to develop DTP than patients with a smaller number of drugs. The possible explanation for this could be polypharmacy might have led to poor-adherence, drug-interactions, and adverse drug events.

In line with other studies [32, 36, 37], patients with older age were more likely to develop DTP than younger patients. This could be explained that patients with older age are more likely to have renal and liver impairment, more co-morbidity and multiple medications. Thus, they could be easily liable to dosing error, drug-interactions, ADR and non-compliance.

In our study, patients with more comorbidity were more likely to experience DTP. This is augmented by a study [29], in which as the number of co-morbidities increased by one unit, the risk of having DTP increased by 28.2%. This might be due to patients with more comorbidities are more likely to have multiple medications. Hence, they could be reluctant to take their medication appropriately. In addition, the risk of drug-interactions and ADR is increased in patients with multiple comorbidities.

Active participation of patients in their care process is important in enhancing the provision of quality pharmaceutical care service and in minimizing problems associated with their treatment [14]. Patients who were not actively involved in their drug therapy decision were approximately four times more likely to have DTPs than those who were actively involved decision-making. In agreement with other studies [37, 38], patients with a negative medication belief were more likely to have DTP than those with a positive belief. The possible explanations for this could be due to patients with negative medication belief are less likely to adhere to their medications. Therefore, in order to minimize DTP, the involvement of patients in decision making and enhancing their positive belief about their medication should be considered in the management process of the HF patients.

### Limitations and strengths

The cross-sectional nature of our study may not provide sufficient evidence of causation about DTP and its contributing factors. Due to self-report concerns, patients may understate socially undesirable activities like medication non-adherence and negative medication beliefs. The results of this study could be affected by the difference in population demographics, health care delivery system, medical practitioner’ expertise and experience, methods of DTP identification and classifications. Therefore, it should be extrapolated to other countries with careful consideration.

Our study has strengths, such as the use of standardized DTPs identification criteria, use of prospective and retrospective patient data, and involvement of a panel of experts in DTP identification. We conducted this study in a teaching and referral hospital which is the largest public hospital in the southwest part of Ethiopia; hence, it could be generalized to other settings in the southwest of Ethiopia.

### Conclusion

DTPs are common among ambulatory care HF patients. Patients with older age, negative medication belief, polypharmacy, co-morbidities and those who were poorly involved in the therapeutic decision were more likely to experience DTP. Despite the traditional prescription refilling, an integrated multidisciplinary approach involving the patients and clinically trained pharmacists should practically be implemented in the medication review and patient monitoring process at ambulatory care clinics in order to improve overall outcomes and reduce drug related complications and associated burdens in HF patients. Furthermore, health care professionals should actively involve patients in their therapeutic decision and educate patients to improve their belief about medication.

## Supporting information

S1 Table Data collection instrument. (DOCX)

S2 Table Dataset. (RAR)

## References

[1] Jessup M, Bozkurt B, Butler J, Casey DE, Drazner MH, Fonarow GC, et al ACCF / AHA Guideline for the Management of Heart Failure A Report of the American College of Cardiology Foundation / American Heart Association Task Force on Practice Guidelines. 2013.

[2] Parker RB, Nappi JM, Cavallari L. Chronic Heart Failure In: Dipro J, Talbert R, Yee G, Matzke G, Wells B, Posey L, editors. Pharmacotherapy A Phathophsyiologic AProach. 9^th^ ed. New York,NY: McGraw-Hill; 2014 p. 278–99.

[3] McMurray JJ, Adamopoulos S, Anker SD, Auricchio A, Böhm M, Dickstein K, et al ESC Guidelines for the diagnosis and treatment of acute and chronic heart failure 2012. Eur Heart J. 2012; 14(8):803–69. 10.1093/eurjhf/hfs105 22828712

[4] Ponikowski P, Anker SD, Alhabib KF. Heart failure: preventing disease and death worldwide. ESC Hear Fail. 2014;1(1):1–39. 10.1002/ehf2.12005 28834669

[5] Cavusoglu Y, Altay H, Ekmekçi A, Eren M, Kucukoglu MS, Nalbantgil S, et al Practical approaches for the treatment of chronic heart failure: Frequently asked questions, overlooked points and controversial issues in current clinical practice. Anatol J Cardiol. 2015; 15(2):1–60. 10.5152/AnatolJCardiol.2015.6767 26574641

[6] Ruppar TM, Delgado JM, Temple J. Medication adherence interventions for heart failure patients: A meta-analysis. Eur J Cardiovasc Nurs. 2015;1–10. 10.1177/1474515115571213 25669661

[7] Fitzgerald AA, Powers JD, Ho PM, Maddox TM, Peterson PN, Allen LA, et al Impact of Medication Nonadherence on Hospitalizations and Mortality in Heart Failure. J Card Fail. 2011;17(8):664–9. 10.1016/j.cardfail.2011.04.011 21807328

[8] Mastromarino V, Casenghi M, Testa M, Gabriele E, Coluccia R, Rubattu S. Polypharmacy in Heart Failure Patients. curent Hear faailure Rep. 2014;11:212–9. 10.1007/s11897-014-0186-8 24493574

[9] Setoguchi S, Choudhry NK, Levin R, Shrank WH, Winkelmayer WC. Temporal Trends in Adherence to Cardiovascular Medications in Elderly Patients After Hospitalization for Heart Failure. Clin Pharmacol Ther. 2010;88(4):548–54. 10.1038/clpt.2010.139 20827266

[10] Murphy GK, Mcalister FA, Eurich DT. Cardiovascular Medication Utilization and Adherence Among Heart Failure Patients in Rural and Urban Areas: A Retrospective Cohort Study. Can J Cardiol. 2015;31(3):341–7. 10.1016/j.cjca.2014.11.024 25633910

[11] Fonarow GC, Albert NM, Curtis AB, Stough WG, Gheorghiade M, Heywood JT, et al Improving Evidence-Based Care for Heart Failure in Outpatient Cardiology Practices Primary Results of the Registry to Improve the Use of Evidence-Based Heart Failure Therapies in the Outpatient Setting (IMPROVE HF). Circulation. 2010; 122:585–96. 10.1161/CIRCULATIONAHA.109.934471 20660805

[12] Schramm TK, Hansen ML, Buch P, Sørensen R, Folke F, Gadsbøll N, et al Persistent Use of Evidence-Based Pharmacotherapy in Heart Failure Is Associated With Improved Outcomes. Circulation. 2007;116:737–44. 10.1161/CIRCULATIONAHA.106.669101 17646585

[13] Europe PC.PCNE Classification for Drug Related Problems. 2010;6.2(20):1–9.

[14] Cipolle R, Strand LM, Morley PC. Pharmaceutical Care Practice. the clinician‟sGuide, 3rd Edition New York,NY: McGraw Hill; 2012.

[15] Dahal P, Kumar SB, Venkataraman R, Fuloria PC. Assessment of clinical pharmacist intervention in tertiary care teaching hospital of southern India. Asian J Pharm Clin Res. 2013;6(2):258–61.

[16] Nivya K, Sri V, Kiran S, Ragoo N, Jayaprakash B, Sekhar MS. Systemic review on drug related hospital admissions–A pubmed based search. Saudi Pharm J. 2015;23(1):1–8. 10.1016/j.jsps.2013.05.006 25685036 PMC4310971

[17] WHO. Promotion of Rational use of medicine care components of Geneva. 2002.

[18] Lu Y, Hernandez P, Abegunde D, Edejer T. The world medicines situation 2011. Medicine expenditures. World Health Organization, Geneva 2011.

[19] Viktil KK, Blix HS. The Impact of Clinical Pharmacists on Drug-Related Problems and Clinical Outcomes. Basic Clin Pharmacol Toxicol. 2008;102(3):275–80. 10.1111/j.1742-7843.2007.00206.x 18248511

[20] Flesch M, Erdmann E. The problem of polypharmacy in heart failure. Current Cardiology Reports. 2006;8:217–25. 17543249 10.1007/s11886-006-0037-7

[21] Gastelurrutia P, Benrimoj SI, Jos E. Negative Clinical Outcomes Associated With Drug-Related Problems in Heart Failure (HF) Outpatients: Impact of a Pharmacist in a Multidisciplinary HF Clinic. J of cardiac Fail. 2011;17(3):217–23. 10.1016/j.cardfail.2010.10.009 21362530

[22] Niquille A, Bugnon O. Relationship between drug-related problems and health outcomes: a cross-sectional study among cardiovascular patients. Pharm World Sci. 2010;32:512–9. 10.1007/s11096-010-9401-1 20526741

[23] Pellicer R, Riu M, Salas E. Patient risk factors for developing a drug-related problem in a cardiology ward. Ther Clin Risk Manag. 2015;11:9–15. 10.2147/TCRM.S71749 25565852 PMC4275111

[24] Granger BB, Ekman I, Hernandez AF, Sawyer T. Results of the Chronic Heart Failure Intervention to Improve Medication Adherence study: A randomized intervention in high-risk patients. Am Heart J. 2015;169(4):539–48. 10.1016/j.ahj.2015.01.006 25819861 PMC5058442

[25] Saheb Sharif-Askari N, Syed Sulaiman SA, Saheb Sharif-Askari F, Hussain AA. Adverse drug reaction-related hospitalisations among patients with heart failure at two hospitals in the United Arab Emirates. Int J Clin Pharm. 2015;37(1):105–12. 10.1007/s11096-014-0046-3 25488317

[26] Yusuff KB, Tayo F. Frequency, types and severity of medication use-related problems among medical outpatients in Nigeria. Int J Clin Pharm. 2011;33:558–64. 10.1007/s11096-011-9508-z 21526413

[27] Ramalho de Oliveira D, Brummel AR. Medication Therapy Management: 10 Years of Experience in a Large Integrated Health Care System. J Manag Care Pharm. 2010;16(3):185–95. doi: 10.18553/jmcp.2010.16.3.185 20331323 10.18553/jmcp.2010.16.3.185 PMC10437567

[28] Jirapermpun A, Pichayapaiboon S. Drug Related Problems in Outpatients with Congestive Heart Failure at Maharat Nakhon Ratchasima Hospital. Nakhon Ratch Med Bull. 2010;34:33–9.

[29] Tegegne GT, Yimamm B, Yesuf EA. Drug Therapy Problems & Contributing Factors Among Patients with Cardiovascular Diseases in Felege Hiwot Referral and Jimma University Specialized Hospital, Ethiopia. Indo Glob J Pharm Sci. 2015;6(1):26–39.

[30] Hsu W, Shen L, Lee C. Drug-related problems vary with medication category and treatment duration in Taiwanese heart failure outpatients receiving case management. J Formos Med Assoc. 2015;(7):1–8. 10.1016/j.jfma.2015.11.014 26774679

[31] Wu JR, Moser DK, Lennie TA, Burkhart P. Medication adherence in patients who have heart failure: a review of the literature. Nurs Clin North Am. 2008;43(1):133–53. 10.1016/j.cnur.2007.10.006 18249229

[32] Tigabu BM, Daba D, Habte B. Drug related problems among medical ward patients in Jimma university specialized hospital, Southwest Ethiopia. J Res Pharm Pract. 2014;3(1):2–6. 10.4103/2279-042X.132702 PMC4078648 24991628

[33] Michaels AD, Spinler SA, Leeper B, Ohman EM, Alexander KP, Newby LK, et al Medication Errors in Acute Cardiovascular and Stroke Patients A Scientific Statement From the American Heart Association. Circulation. 2010;121(14):1664–82. 10.1161/CIR.0b013e3181d4b43e 20308619

[34] Koh Y, Kutty FB, Li SC. Drug-related problems in hospitalized patients on polypharmacy: the influence of age and gender. Ther Clin Risk Manag. 2005;1(1):39–48. 18360542 10.2147/tcrm.1.1.39.53597 PMC1661606

[35] Snyder ME, Frail CK, Jaynes H, Pater KS, Zillich AJ. Predictors of Medication-Related Problems among Medicaid Patients Participating in a Pharmacist-Provided Telephonic Medication Therapy Management Program. Pharmacotherapy. 2014;34(10):1022–1032. 10.1002/phar.1462 25051943 PMC4426336

[36] Ogbonna BO, Ezenduka CC, Opara CA, Ahara L. Drug Therapy Problems in Patients with Type-2 Diabetes in a Tertiary Hospital in Nigeria. Int J Innov Res Dev. 2014;3(1):494–502.

[37] Horne R, Weinman J. Patients ‘ beliefs about prescribed medicines and their role in adherence to treatment in chronic physical illness. Saudi Pharm J. 1999;47(6):555–67. 10.1016/s0022-3999(99)00057-4 10661603

[38] De Smedt RH, Denig P, van der Meer K, Haaijer-Ruskamp FM, Jaarsma T. Self-reported adverse drug events and the role of illness perception and medication beliefs in ambulatory heart failure patients: A cross-sectional survey. Int J Nurs Stud. 2011;48(12):1540–50. 10.1016/j.ijnurstu.2011.05.014 21774932

