# Supplementary material for: Drug therapy problems and contributing factors in the management of heart failure patients in Jimma University Specialized Hospital, Southwest Ethiopia
Source: PLoS One. 2018 Oct 23;13(10):e0206120. doi: 10.1371/journal.pone.0206120 (PMC6198973; doi:10.1371/journal.pone.0206120)
Supplement: S1 Table — (DOCX) [file pone.0206120.s001.docx]

**Data collection tool**

**Jimma University**

**College of Health Sciences**

**School of Pharmacy**

**Annexes**

**Annex I: Patient Information Sheet**

**Name of the principal investigator:** **Yirga Legesse**

**Name of study area:** JUSH

**Research budget covered by:** Jimma University

**Research objective:** To assess the pattern, prevalence and determinants of drug-therapy problems in the management of heart failure patients in chronic follow up in JUSH

**Significance of the study:** The outcome of this study will provide baseline information for future researchers and it will improve the awareness of health care professionals and policy makers about importance of pharmaceutical care practice. Hence, it will contribute to the formulation and implementation of pharmaceutical care services in the health care system policy

**Study procedure:** Patient specific data will be collected using structured data collection tool to determine if the patient's drug-related needs are being met; i.e. all the patient's medications are appropriately indicated, the most effective available and the safest possible agent is used, and the patient is able and willing to take the medication as intended

**Risks:** No risks except the time that patient spend during the interview.

**Participant right:** The patient has a right to stop the interview at any time, or to skip any question that he/she does not want to answer.

**Benefit:** The study is beneficial for the patient in improving quality of service delivery in future visits. It informs health care providers about the status of care. It also can be used as a source of information for the hospital and policy makers.

**Incentives:** You will not be provided any specific incentive for taking part in the research other than acknowledgment.

**Confidentialities:** The study result will not include patient’s name and address and any information communicated will be kept confidential.

**Agreement:** Patients are expected to be fully voluntary to participate in the study.

**Whom to contact:** If you have any kind of inconveniencies about the study, you can contact the following individual: Yirga Legesse: Cell Phone: 0914861882 or [yirga.pharma@gmail.com](mailto:yirga.pharma@gmail.com).

**Annex II: Informed Consent**

**Research title:** Drug Therapy Problems and Contributing Factors among ambulatory heart failure patients on chronic follow up in Jimma University Specialized Hospital, Southwest Ethiopia

Card number______________ Code number_____________________

1. I confirm that I understand the information sheet for the above study and have had the opportunity to ask questions.
2. I understand that my participation is completely voluntary and that I am free to
 withdraw at any time, without giving any reason, without my medical care or legal
 rights being affected.
3. I understand that my medical notes will be looked at by data collectors of this study
 and necessary information will be extracted. I give permission for these individuals to
 have access to my records.
4. I agree to take part in the above study. I would like to confirm my agreement by
 signing.

Participant’s name _______________________Signature_______ date_______

Name of the data collector: _________________ Signature: ______ date________
Name of the principal investigator: ____________Signature: ______ date________

Thank you for your participation and cooperation

**Greetings!**
My name is Sr. / Ato -----------------------------------------------------

I am data collector for master student Yirga Legesse currently working his research work for graduation in Clinical Pharmacy in Jimma University, College of Health Sciences, and Department of Pharmacy.

The objective of the research is to assess drug therapy problems and contributing factors among heart failure patients in Jimma University Specialized Hospital.

I would like to assure you that the study is confidential and secure I will not keep a record of your name and address. You have a right to stop the interview at any time, or to skip any question that you do not want to answer. Your correct answer to the questions can make the study achieve its goals. Therefore, you are kindly requested to respond genuinely and voluntary with patience. The interview may take few minutes. I would greatly appreciate your help in responding to this study.

Result of the interview:

1. Completed

2. Partially completed

3. The interviewee refused

4. Others____________

**Annex III: Data collection tool**

**Jimma University**

**College of Health Sciences**

**School of Pharmacy**

**Data collection tool to identify drug therapy problems and associated factors among heart failure patients in JUSH**

1. **Questionnaire English Version**
2. **Participants’ Scio demographic Characteristics and disease related questions**

| **No** | **Questions** | **Response** |
| --- | --- | --- |
|  | Patient’s sex | 🖵 Male 🖵Female |
|  | Age | ______years |
|  | Body weight | _______kg |
|  | Marital status | 1. Single🖵 2. Married 🖵 3. Divorced 🖵 4. Widowed 🖵 |
|  | Religion | 1. Orthodo 🖵 2. Protestant🖵 3. Muslim 🖵 4. Others-------- |
|  | Educational status | 1. can’t read and write 🖵 2. Non-formal education 🖵 3. Primary education (1-8 grade) 🖵 4. Secondary education (9-12 grade) 🖵 5. Tertiary education (diploma and above) 🖵 |
|  | Occupation | _____________ |
|  | Monthly income in Ethiopian Birr? | _____________ Birr |
|  | Alcohol consumption | Yes: No: |
|  | Cigarette smoking status | Yes: No: |
|  | khat chewing status | Yes: No: |
|  | Traditional and herbal medicine use | Yes: No: |
|  | Living status: | 1. living with famly 2. living with friends 3. Lives alone 4. Others-- |
|  | Residence | rural urban |
|  | Number of hospitalizations since the last one year | Zero times  one times  two times  ≥ three times |
|  | Duration on treatment | __________ |
|  | Time since diagnosis | __________ |

1. **Assessment of Patient’s perceptions /beliefs and concerns about their medicines**
   1. Do you belief that your health, at present, depends on your medicines?

- Strongly agree agree uncertain disagree strongly disagree
  1. Do you belief that your life would be impossible without your medicines?

Strongly agree agree uncertain disagree strongly disagree

- 1. Do you belief that without your medicines you would become very ill?

Strongly agree agree uncertain disagree strongly disagree

- 1. Do you belief that your health in the future will depend on your medicines

Strongly agree agree uncertain disagree strongly disagree

- 1. Do you belief that your medicines protect you from becoming worse?

Strongly agree agree uncertain disagree strongly disagree

- 1. Do you belief that Having to take medicines worries you?

Strongly agree agree uncertain disagree strongly disagree

- 1. Do you sometimes worry about the long-term effects of your medicines

Strongly agree agree uncertain disagree strongly disagree

- 1. Do you belief that your medicines are a mystery to you?

Strongly agree agree uncertain disagree strongly disagree

- 1. Do you think that your medicines disrupt your life?

Strongly agree agree uncertain disagree strongly disagree

- 1. Do you sometimes worry about becoming too dependent on your medicines?

Strongly agree agree uncertain disagree strongly disagree

1. **Assessment of Patients experience about health care service and their relationship with their providers**
   1. Did your health care provider listen to you and treat your concerns seriously?

At all times Most of the time sometimes rarely never

- 1. Do you feel that the service has helped you to better understand and address your difficulties?

At all times Most of the time sometimes rarely never

- 1. Did you get involved in making choices about your treatment and care?

At all times Most of the time sometimes rarely never

- 1. Did you have confidence in your health care provider and his /her skills

At all times  **M**ost of the time  **s**ometimes  **r**arely never

- 1. Did your medicines are available in the health institution where you receive care?

At all times  **M**ost of the time  **s**ometimes  **r**arely never

- 1. Did you get adequate counseling about your medicines?

At all times  **M**ost of the time  **s**ometimes  **r**arely never

- 1. Did you communicate freely all about your problems with your health care provider

At all times  **M**ost of the time  **s**ometimes  **r**arely never

- 1. Were you given information about options for choosing a treatment appropriate for your problems?

At all times  **M**ost of the time  **s**ometimes  **r**arely never

- 1. How satisfied are you with the type of treatment that you received?

Very satisfied satisfied not sure Dissatisfied Very dissatisfied

- 1. How satisfied are you with the overall experience of using this service?

Very satisfied satisfied not sure Dissatisfied Very dissatisfied

1. **Data abstraction format /checklist (data from review of patient’s medical record)**
   1. **Card no------- age----------- sex---- weight ---- height-------**
   2. **Chief compliant ----------------------------------------------------------------------------------**
   3. **History of present illness ---------------------------------------------------------------------------------------------------------------------------------------------------------------------------------------------------------------------------------------------------------------------------------------------------------------------------------------------------------------------------------------------------------------------------------------------------------------------------------------------------------------------------------------------------------------------------------------------------------------------------------------------**
   4. **Initial date of diagnosis ------------------------**
   5. **Duration on treatment----------------------------**
   6. **Documented/suspected ADR/Allergy if any-----------------------------------------------------------------------------------------------------------------------------------------------------------------**
   7. **Past medical history** (relevant illnesses, hospitalizations, surgical procedures, emergency department visits, injury)----------------------------------------------------------------------------------------------------------------------------------------------------------------------
   8. **Past medication/immunization history**

| Indication | Drug therapy | Response | Year/month /date |
| --- | --- | --- | --- |
|  |  |  |  |
|  |  |  |  |
|  |  |  |  |
|  |  |  |  |
|  |  |  |  |

- 1. **Vital signs:**

| **Date** |  |  |  |  |  |  |  |  |  |  |  |  |  |
| --- | --- | --- | --- | --- | --- | --- | --- | --- | --- | --- | --- | --- | --- |
| BP |  |  |  |  |  |  |  |  |  |  |  |  |  |
| HR |  |  |  |  |  |  |  |  |  |  |  |  |  |
| RR |  |  |  |  |  |  |  |  |  |  |  |  |  |
| T^0^ |  |  |  |  |  |  |  |  |  |  |  |  |  |

- 1. **Relevant laboratory results and investigations**

| **Date** |  |  |  |  |
| --- | --- | --- | --- | --- |
| **LFT:** |  |  |  |  |
| **RFT:** |  |  |  |  |
| **CBC:** |  |  |  |  |
| **RBS/FPG** |  |  |  |  |
| **Lipid Profile:** |  |  |  |  |
| **Echocardiography:** |  |  |  |  |
| **ECG:** |  |  |  |  |
| **Electrolyte** |  |  |  |  |
| **Ttroponi/CK-MB** |  |  |  |  |
| **Ultrasound** |  |  |  |  |
| **X-Ray (**type, reading, Interpretation): |  |  |  |  |
| **Others** |  |  |  |  |

- 1. **Medical conditions and medications**

| **Date of visit** | **Working diagnosis** | **Date of medication ordered** | **Drug product** | **Dosage regimen**  (dose, route, frequency, duration) |
| --- | --- | --- | --- | --- |
|  |  |  |  |  |
|  |  |  |  |  |
|  |  |  |  |  |
|  |  |  |  |  |
|  |  |  |  |  |
|  |  |  |  |  |
|  |  |  |  |  |
|  |  |  |  |  |
|  |  |  |  |  |
|  |  |  |  |  |

1. **Drug therapy problem Identification and assessment: Logical questions to identify whether or not the patient is experiencing a drug therapy problem**
2. **Is the medication (indication) appropriate?**
   1. **Unnecessary drug therapy**
3. Is there no valid medical indication for the drug therapy at this time?
4. Do multiple drug products are being used for a condition that requires single drug therapy?
5. Is the medical condition more appropriately treated with nondrug therapy?
6. Does drug therapy is being taken to treat an avoidable adverse reaction associated with another medication?
7. Is drug abuse, alcohol use, or smoking causing the problem?
   1. **Need for additional drug therapy**
8. Does a medical condition require the initiation of drug therapy?
9. Does Preventive drug therapy required to reduce the risk of developing a new condition?
10. Does a medical condition require additional pharmacotherapy to attain synergistic or additive effects?
11. **Is the drug therapy effective for the disease condition?**
    1. **Ineffective drug therapy**
12. Is the drug not the most effective for the medical problem?
13. Is the medical condition refractory to the drug product?
14. Is the dosage form of the drug product inappropriate?
15. Is the drug product not an effective product for the indication being treated?
    1. **Dosage too low**
16. Is the dose too low to produce the desired response?
17. Is the dosage interval too infrequent to produce the desired response?
18. Does a drug interaction reduce the amount of active drug available?
19. Is the duration of drug therapy too short to produce the desired response?
20. **Is the drug therapy as safe as possible?**
    1. **Adverse drug reaction**
21. Does the drug product cause an undesirable reaction that is not dose-related?
22. Is a safer drug product required due to risk factors?
23. Does a drug interaction cause an undesirable reaction that is not dose-related?
24. Was the dosage regimen administered or changed too rapidly?
25. Does the drug product cause an allergic reaction?
26. Is the drug product contraindicated due to risk factors?
27. Is there any history of allergy of the prescribed drug?
    1. **Dosage too high**
28. Is dose too high?
29. Is the dosing frequency too short?
30. Is the duration of drug therapy too long?
31. Does a drug interaction occur resulting in a toxic reaction to the drug product?
32. Was the dose of the drug administered too rapidly?
33. **Does the patient comply with the prescribed medication regimen?**
    1. **Non-compliance**
    2. Doesn’t the patient understand the instructions?
    3. Does the patient prefer not to take the medication?
    4. Does the patient forget to take the medication?
    5. Is drug product too expensive for the patient?
    6. Can’t the patient swallow or self-administer the drug product appropriately?
    7. Is the drug product not available for the patient?

**Questionnaire Amharic Version**

**የታካሚ መረጃ ወረቀት**

**የተመራማሪ ስም: ይርጋ ለገሰ**

**ጥናት የሚካሄድበት ቦታ:** ጂማ ዩኒቨርስቲ ስቴሻላይዝድ ሆስፒታል

**የትናቱ በጀት ከፋይ:** ጂማ ዩኒቨርስቲ

**የትናቱ አላማ:** በተመላላሽ የልብ በሽቶኞች ህክምና ላይ ያሉ ከመድሀኒት የተያያዙ ችግሮች መገምገም እና ወሳኝ ነገሮችን መለየት።

**የጥናቱ መቀሜታ:**ጥናቱ ለጤና ባለሞያዎቸ እና ለፖሊሲ አውጪዎች ስለመድሃኒት አጠቃቀም እና ችግሮቻቸው ያላቸው ትኩረት እነዲጨምር ያረጋል። በተጨማሪም በተመሳሳይ ዙርያ ወደፊት ለሚሰሩ ትናቶች መሰረታዊ መረጃ ይሰጣል።

**የጥናቱ ሂደት:** መረጃ ሰብሳቢ ሰዎች የተሳታፊውን ፍቃድ ካገኙ በኋላ በማስጠየቅያ ወረቀት ቃለመጠይቅ ያረጋሉ።ከዛ በመቀጠል ከመዝገብ ካርዱ የተመዘገበውን መረጃ ይወስዳሉ።

**ጉዳት:** ጥናቱ ምንም አይነት ጉዳት የለውም።

**የተሳታፊ መብት:**ተሳታፊው ቃለመጠይቁን በፈለገው ሰአት ማቋረጥ እንዲሁም ያልፈለገውን ጥያቄ አለመመለስ ይችላል።.

**ጥቅም:**ጥናቱ ለቀጣይ ግዜ ጥራት ያለውን አገልግሎት ለመስጠት ይጠቅማል።

**ማበረታቻ:** በጥናቱ ላይ ተሳታፊ በመሆን የሚሰጥ ምንም አይነት ማበረታቻ የለውም።.

**ምስጢር ጠባቂነት:**ጥናቱ የተሳታፊውን ስም፣አድራሻ እና ሌላ ያስተላለፈውን መልእክት በተገቢው መልክ ይጠብቃል።

**ስምምነት:** ታካሚው በሙሉ ፍላጎት ተሳታፊ እንደሚሆን ይጠበቃል።.

**ለተጨማሪ መረጃ የዚህ ጥናት አስተባባሪ ማነጋገር ይችላሉ።**

1. ይርጋ ለገሰ: የጥናቱ ተመራማሪ

- ስልክ: 0914861882
- ኢሜል: yirg.pharma[@gmail.com](mailto:s.weldegebreal@gmail.com)

**በጥናቱ ለሚሳተፉ የስምምነት ማረጋገጫ**

እኔ ስለዚህ ጥናት ሙሉበሙሉ ተነግሮኝ ተረድቻለሁ።አላማውም በተመላላሽ የልብ ህመምተኞች ህክምና ላይ ያሉ ከመድሀኒት የተያያዙ ችግሮች መገምገም እና ወሳኝ ነገሮችን መለየት።

ይህ ተሳትፎ በፈቃደኝነት ላይ የተመረኮዘ መሆኑን ተረድቼለሁ።በተጨማሪም የተረዳሁት በዚህ ተሳትፎየ አገኗኛለሁ የምለው ምንም አይነት የተለየ አገልግሎት: ክፍያ ወይም ስጦታ እንደማይኖር ተረድቻለሁ።

ይህ ውል የሚያገለግለው ለዚህ ብቻ ነው።

ከዚህ በታች ስሜ ያለው በዚህ ጥናት ለመሳተፍ ተስማምቼለሁ።

የተሳታፊ ስም_____________ ፊርማ_______ ቀን__________

የመረጃ ሰብሳቢ ስም: _________________ : ፊርማ ________ ቀን________
የተመራማሪ ስም: _________________ ፊርማ: ________ ቀን________

ለተሳትፎችሁ እና ድጋፋቹሁ አመሰግናለሁኝ!

**ስሜ:አቶ/ወ/ሪት**____________

እኔ የይርጋ ለገሰ የሚባል በአሁን ሰአት ለምረቃ ማስተርስን የሚሰራ መረጃ ሰብሳቢ ነኝ።

የጥናቱ ዋና አላማ በተመላላሽ የልብ በሽቶኞች ህክምና ላይ ያሉ ከመድሀኒት የተያያዙ ችግሮች መገምገም እና ወሳኝ ነገሮችን መለየት ነው።

ጥናቱ ምስጥር ጠባቂ መሆኑን ላረጋግጥላቹ እወዳለሁኝ ቃለመጠይቁን በፈለጋችሁት ሰአት ማቋረጥ ትችላላችሁ በዚህ ጥናት ምላሽ በመስጠት በማገዛችሁ በጣም አመሰግናለሁኝ።

የቃለመጠይቁ ውጤት

አልቀዋል____________ በከፊል አልቀዋል____________

ሌላ____________ ተነፍገዋል ____________

**ሀ. የታካሚ ማህበረሰባዊ ጥያቄዎች/ባህርያቶች**

**መምርያ**: በተዘጋጀውን √ ሳጥን ምልክትያድርጉ በተጨማሪ ታካሚውን በመጠየቅ ክፍት ቦታውን ይምሉ።

| ተራቁጥር | ጥያቄዎች |  |
| --- | --- | --- |
|  | የታካሚ ጾታ | ወንድ🖵  ሴት🖵 |
|  | እድሜህ ስንትነው? | ____________አመት |
|  | የሰብነት ክብደት | _______kg |
|  | የጋብቻሁኔታ | 1. ያላገባ🖵 2. ያገባ🖵 3. የተፋታ🖵 4. ባሏ/ሚስቱየሞተባት/በት🖵 |
|  | ሃይማኖትህምንድነው? | 1. ኦርቶዶክስ🖵 2. ፕሮቲስታንት🖵 3. ሙስሊም🖵 4. ሌሎች____________ |
|  | ስራ ህምንድን ነው? | ------------------------ |
|  | ከፍተኛ የትምህርት ደረጃህ ስንትነው? | 1. ማንበብ እና መጻፍ አልችልም 2. መደበኛየትምህርትደረጃየለኝም🖵 3. ከ1_8🖵 4. ከ9᎐12🖵 5. ዲፕሎማእናከዛበላይ🖵 |
|  | ወርሃዊ ገቢህ ስንትነው? | _______________ብር |
|  | አልኮሆል ትጠጣለህ? | 🖵አዎ 🖵አይደለም |
|  | ሲጋራማ ታጨሳልህ ? | 🖵አዎ 🖵አይደለም |
|  | ጫት ትቅማለህ ? | 🖵አዎ 🖵አይደለም |
|  | ባህላዊመድሃት ወስደህ ታቃለህ? | 🖵አዎ 🖵አይደለም |
|  | ማን ጋር ነው ምትነረው? | 1. ከቤተሰብ 2. ከጎረቤት 3. ብቻየነውምነሮው 4. ሌላ------ |
|  | መኖርያ | ገጠር ከተማ |
|  | ከባለፈው አመት ወደዚ ያለውስንት ግዜ ሆስፒታል ተኝተዋል | ምንም  አንድ ግዜ  ሁለት ግዜ  ≥ ሶስት ግዜ |
|  | በሽታዎ በምርመራ ከተረጋገጠ ስንት አደረገ |  |
|  | ህክምና ከጀመሩ ስንት አደረጉ |  |

**ለ.ታካሚዎቸች ስለሚወስዱት መድሐኒት ያላቸው አመለካከት ግምገማ**

- 1. በአሁኑ ሰዐት ጤናዎ በመድሐኒትዎ የተመሰረተ ነው ብሎ ያምናሉ

በጣም እስማማለሁ እስማማለሁ እርግጠኛአደለሁም አልስማማም በጣምአልስማማም

- 1. ያለመድሐኒትዎ መኖር አልችልም ብሎ ያምናሉ

በጣምእስማማለሁ እስማማለሁ እርግጠኛአደለሁም አልስማማም በጣምአልስማማም

- 1. መድሐኒት ካልወሰድኩ በጣምያመኛል ብሎ ያምናሉ

በጣም እስማማለሁ እስማማለሁ እርግጠኛ አደለሁም አልስማማም በጣምአልስማማም

- 1. የወደፊት ጤናዎ በመድሐኒትዎ የተመሰረተ ነው ብሎ ያምናሉ

በጣም እስማማለሁ እስማማለሁ እርግጠኛ አደለሁም አልስማማም በጣምአልስማማም

- 1. መድሐኒትዎ በሽታዎ እንዳይባባስ ይከላከልኛል ያምናሉ

በጣም እስማማለሁ እስማማለሁ እርግጠኛአደለሁም አልስማማም በጣምአልስማማም

- 1. መድሐኒት ሁሌ መውሰድ ያሳስቦታል/ያሰጨንቆታል

በጣምእስማማለሁ እስማማለሁ እርግጠኛአደለሁም አልስማማም በጣምአልስማማም

- 1. አንዳንዴ የመድሐኒትዎ የወደፊት የረዥም ግዜ ጉዳት ያሳስቦታል

በጣም እስማማለሁ እስማማለሁ እርግጠኛአደለሁም አልስማማም በጣምአልስማማም

- 1. መድሐኒትዎ ለእርስዎ ድብቅ/ሚሰጥር ነው ብሎ ያምናሉ

በጣም እስማማለሁ እስማማለሁ እርግጠኛአደለሁም አልስማማም በጣምአልስማማም

- 1. መድሐኒትዎ በኑሮዎ ለይችግር እየፈጠሮቦትነው

በጣምእስማማለሁ እስማማለሁ እርግጠኛአደለሁም አልስማማም በጣምአልስማማም

- 1. አንዳንዴ በጣም የመድሐኒተ ጥገኛ ስለመሆን ያሳስቦታል

በጣምእስማማለሁ እስማማለሁ እርግጠኛአደለሁም አልስማማም በጣምአልስማማም

**ሐ. ታካሚዎቸ ችስለም ያገኙት የጤና አገልግሎት አስተያየት እና ከም ያገለግልዋቸው የጤና ባለሞያዎች ያላቸው ግኑኘት ግምገማ**

1. ሐኪምዎ ስለ ሚፈልጉት ነገር ትኩርት ሰጦ ያዳምጦታል ?

ሁሉገዜ አብዛኛውግዜ አንዳአንድግዜ አልፎአልፎ በፍጹም

1. የምያገኙት አገልግሎት ስለችግርዎ በበለጠ እንዲረዱት እንደአገዞት ይሰሞታል?

ሁሉገዜ አብዛኛውግዜ አንዳአንድግዜ አልፎአልፎ በፍጹም

1. በሚደረግሎት ህክምና/መድሀኒት ምርጫውሳኔ ላይእንዲሳተፉ ይደረጋል ?

ሁሉገዜ አብዛኛውግዜ አንዳአንድግዜ አልፎአልፎ በፍጹም

1. አገልግሎት ከሚሰጦት የጤና ባለሙያ እና ክህሎቶቹን እምነት አሎት

ሁሉገዜ አብዛኛውግዜ አንዳአንድግዜ አልፎአልፎ በፍጹም

1. የታዘዘሎት መድሐኒት አገልግሎት ከምያገኙበት የጤናተቃም ያገኛሉ?

ሁሉገዜ አብዛኛውግዜ አንዳአንድግዜ አልፎአልፎ በፍጹም

1. ሰለምወስዱት መዳሐኒት በቂየሆነ ምክርይ ሰጦታል

ሁሉገዜ አብዛኛውግዜ አንዳአንድግዜ አልፎአልፎ በፍጹም

1. ከሐኪምዎ ስለ ችግርዎ ሁሉ በነጻነት ይነጋገራሉ

ሁሉገዜ አብዛኛውግዜ አንዳአንድግዜ አልፎአልፎ በፍጹም

1. ስለምታገኘው የህክምና/መድሃኒትአይነት ከአንድ በላይ ምርጫ ራስህን እንድትመርጥ አማራጭ ተሰጦህያቃል

ሁሉገዜ አብዛኛውግዜ አንዳአንድግዜ አልፎአልፎ በፍጹም

1. እያገኙት ባለ ህክምና/መድሀኒት ዐይነት እረካታዎ እንዴትነው?

*በጣምረክቻለሁ ረክቻለሁ እርግተኛአደለሁም አልረካሁም በጣምአልረካሁም*

1. እያገኙት ባለ አገልገሎት ሁሉ እረካታዎ እነዴትነው?

*በጣምረክቻለሁ ረክቻለሁ እርግተኛአደለሁም አልረካሁም በጣምአልረካሁም*
